# Exploring Mycosporine-like Amino Acid UV-Absorbing Natural Products for a New Generation of Environmentally Friendly Sunscreens

**DOI:** 10.3390/md21040253

**Published:** 2023-04-19

**Authors:** Nedeljka Rosic, Mike Climstein, Glen M. Boyle, Duy Thanh Nguyen, Yunjiang Feng

**Affiliations:** 1 Faculty of Health, Southern Cross University, Gold Coast, QLD 4225, Australia; 2 Marine Ecology Research Centre, Southern Cross University, Lismore, NSW 2480, Australia; 3 Physical Activity, Sport and Exercise Research (PASER) Theme, Faculty of Health, Southern Cross University, Gold Coast, QLD 4225, Australia; 4 Physical Activity, Lifestyle, Ageing and Wellbeing, Faculty Research Group, Faculty of Health Sciences, The University of Sydney, Sydney, NSW 2000, Australia; 5 Cancer Research Program, QIMR Berghofer Medical Research Institute, Brisbane, QLD 4006, Australia; 6 School of Biomedical Sciences, Faculty of Health, Queensland University of Technology, Brisbane, QLD 4000, Australia; 7 School of Biomedical Sciences, Faculty of Medicine, University of Queensland, Brisbane, QLD 4072, Australia; 8 Griffith Institute for Drug Discovery, Griffith University, Brisbane, QLD 4111, Australia

**Keywords:** mycosporine-like amino acids, antioxidants, ultraviolet-absorbing compounds, sunscreen, bioactivity, bioproduct screening, omics, green technology

## Abstract

Human skin needs additional protection from damaging ultraviolet radiation (UVR: 280–400 nm). Harmful UVR exposure leads to DNA damage and the development of skin cancer. Available sunscreens offer chemical protection from detrimental sun radiation to a certain extent. However, many synthetic sunscreens do not provide sufficient UVR protection due to the lack of photostability of their UV-absorbing active ingredients and/or the lack of ability to prevent the formation of free radicals, inevitably leading to skin damage. In addition, synthetic sunscreens may negatively affect human skin, causing irritation, accelerating skin aging and even resulting in allergic reactions. Beyond the potential negative effect on human health, some synthetic sunscreens have been shown to have a harmful impact on the environment. Consequently, identifying photostable, biodegradable, non-toxic, and renewable natural UV filters is imperative to address human health needs and provide a sustainable environmental solution. In nature, marine, freshwater, and terrestrial organisms are protected from harmful UVR through several important photoprotective mechanisms, including the synthesis of UV-absorbing compounds such as mycosporine-like amino acids (MAAs). Beyond MAAs, several other promising, natural UV-absorbing products could be considered for the future development of natural sunscreens. This review investigates the damaging impact of UVR on human health and the necessity of using sunscreens for UV protection, specifically UV-absorbing natural products that are more environmentally friendly than synthetic UV filters. Critical challenges and limitations related to using MAAs in sunscreen formulations are also evaluated. Furthermore, we explain how the genetic diversity of MAA biosynthetic pathways may be linked to their bioactivities and assess MAAs’ potential for applications in human health.

## 1. Introduction

The levels of ultraviolet (UV) radiation have continued to increase over the past century [1]. Excessive exposure to UV radiation has been associated with the development of the majority of skin cancers [2,3]. Based on epidemiologic data, the development of melanoma and basal cell carcinoma (BCC) was associated with excessive sun exposure resulting in sunburns [4]. On the other hand, the development of squamous cell carcinoma (SCC) has been associated with a lifetime of prolonged sun exposure, as seen in the example of occupational sun exposure [5]. The negative UVR effect on the skin occurs via mutagen impact on DNA, resulting in the formation of dimeric photoproducts between pyrimidine bases (so-called cyclobutane pyrimidine dimers (CPDs)), leading to DNA base damage [6]. However, different components of UV radiation have varying impacts on the skin, with longer wavelength ultraviolet A (UVA) having a higher penetrance, reaching the dermis skin layer. Alternatively, shorter wavelength ultraviolet B (UVB) mainly impacts the epidermal layer [7]. Importantly, both UV components, UVA and UVB, that reach the Earth’s surface can damage DNA directly and indirectly via reactive oxygen species (ROS), although UVB has a higher mutagenic and carcinogenic impact [3,8].

In nature, organisms have developed different mitigation strategies to protect from damaging UV radiation. Natural products such as mycosporine-like amino acids (MAAs) are major UV-protective agents found in various species living in marine and freshwater environments, including symbiotic and nonsymbiotic species [8,9,10,11,12,13,14]. Marine organisms exposed to severe changes in light irradiance can adapt via photoacclimation [15,16], although they become more susceptible to additional external stressors such as temperature and acidification [15,16]. The mitigation strategies of organisms like corals and sea urchins included the accumulation of more MAAs in tissues exposed to higher UVR [17]. MAAs are UV-absorbing secondary metabolites, which are found to be most commonly represented among aquatic species. These small molecules are involved in photoprotection and reducing UV-induced damage and osmoregulation, and they show promising therapeutic potential [18]. The photoprotective capacities of MAAs are based upon their ability to absorb light in the UV range, including UVA in the range of 315–400 nm and UVB in the range of 280–315 nm, with absorption maxima occurring in the range of 310–362 nm [19,20,21]. There are substantial variations in the composition of MAAs, resulting in variability in species’ UV-absorbing profile [22,23,24], indicating diversity in their UV-absorbing capacity, which is also influenced by seasonal UV fluctuations [25,26,27], environmental stress [20,28,29,30], and nutrient, specifically nitrogen, availability [31,32,33,34].

MAAs are characterized by a high molar extinction coefficient of ε = 28,100–50,000 M^−1^ cm^−1^ and the ability to disperse absorbed radiation as heat without the production of free radicals [12,35,36]. Beyond the photoprotective role, MAAs demonstrate antioxidative capacity and can scavenge ROS produced in cells to prevent further DNA damage [37,38,39,40,41,42]. Antioxidants (synthetic or coming from natural resources) are commonly used in modern medicine as bioactive compounds due to their ability to decrease the number of free radicals in cells and tissues [43]. Furthermore, MAAs demonstrate additional biotechnological potentials, including anti-inflammatory, anti-proliferative, and anti-aging properties [44,45,46]. The promising pharmacological properties of MAAs could be further utilized in various biotechnological applications, such as more efficient skin UV protection, which is important for improved skin cancer prevention.

## 2. Fitzpatrick Phototype and UV Protection Strategies

The skin is the largest organ in the human body, representing approximately 16% of body mass [3]. Higher levels of UV exposure have been associated with increased skin cancer prevalence in humans [47]. Therefore, besides internal UV protection, human skin requires additional external mechanisms to reduce sun-induced DNA damage and potential skin cancer formation. A number of UV-absorbing compounds (i.e., UV-absorbing pigments and other molecules) were found to provide internal UV protection, including additional mechanisms such as increased epidermal thickness, DNA repair mechanisms, and the accumulation of antioxidants [32,48]. For example, the human pigment melanin has a major photoprotective role in reducing sun-induced cancer and function as an antioxidant by scavenging free radicals produced during UVR exposure [48,49,50]. This pigment has two main forms: eumelanin, which is highly UV-protective, and pheomelanin, which has a lower UV-protective capacity [48]. Though the pigment melanin has a strong UV-protective capacity, it does not provide complete protection, but protecting against approximately 50–70% of UVR [48]. People with less eumelanin are more UV-sensitive [3]. Differences in skin pigmentation that impact UV risks are scaled based on the Fitzpatrick scale (Table 1). The Fitzpatrick skin type scale evaluates UV risk and UV exposure tolerance levels, indicating differences in required skin protection. Skin complexion is recognized as one of the most important determinants of UV sensitivity and the risk of developing skin cancer [3]. He and colleagues [51] described the Fitzpatrick skin phototype (skin, eyes, and hair pigmentation) classification system as the most common method to assess sunburns and the subsequent risk of developing skin cancer, primarily basal cell carcinoma (BCC) and malignant melanoma [52]. Consequently, different external sun protection strategies have been recommended depending on the skin phototype (Table 1) [3].

As internal mechanisms for UV protection are often insufficient to prevent UV skin damage, a number of external strategies are used. Different types of strategies are often applied to increase the amount of protection from damaging UVR, including chemical barriers (i.e., sunscreens) and physical barriers (i.e., UV protection clothing, hats, and shade). A large-scale randomized control study with 1600 participants completed in Australia found that the incidence of squamous cell carcinoma and melanoma was significantly reduced in individuals who used sunscreen daily as compared to individuals who used sunscreen on a discretionary basis [53]. Daily use of sunscreen reported a significant decrease (rate ratio 0.62) in actinic keratosis, which is a precursor to the development of squamous cell carcinoma, as compared to controls [54]. Sunscreens were found to be more efficient in reducing skin cancer prevalence compared to UV-protective clothing [55,56], although in some cases, UV-protective clothing was the preferred option [57].

## 3. Natural UV-Absorbing Compounds

Current chemical protection from UVR is inadequate because synthetic sunscreen products contain active ingredients that may lack photostability [58,59,60]. The photostability of many commonly used chemical UV filters (e.g., oxybenzone, benzophenone-3, which is permitted up to 6% in sunscreen formulations [30]) was tested individually and in combination with other active ingredients [61]. The majority of these compounds showed poor photostability due to photochemical reactions, such as trans-cis isomerization or keto-enol tautomerism, or due to reactions with other UV filters, which produce byproducts [61,62]. Synthetic sunscreens can also negatively impact human health, causing photosensitization and photo irritation, resulting in allergic reactions, free radical formation leading to skin damage, skin irritation, and skin aging acceleration [58,59,60]. Several approaches have been applied during the last decade to improve the photostability of synthetic UV filters, including the use of antioxidants, encapsulation, and the addition of quenching molecules to the sunscreen formulation [63]. For example, avobenzone, a commonly used UVA filter with a very high number of photodegradation products, showed improved photostability in the presence of Vitamins A and C and ubiquinone within the formulation, resulting in an improved SPF value [64]. However, negative environmental impact coming from the application of different synthetic sunscreens remains a problem, as reported in animal and human studies, including the neurotoxic effect of some sunscreen active ingredients [59], endocrine disruption, malformations, coral bleaching, and other detrimental impacts on ecosystems [63,65]. The major issue for these UV filters is their long retention in the environment, slow degradation, and possibly toxicity [58,59]. Consequently, there has been a shift in industry interest toward the use of natural, environmentally friendly UV-absorbing products as UV filters that are biocompatible, biodegradable, and have no toxic properties [63,66].

Natural products (NPs) have become increasingly popular in the development of sunscreens due to their ability to provide a broad spectrum of UV protection and their advantages over synthetic compounds. These small molecules are derived from natural sources such as medicinal plants, herbs, fungi, and marine organisms, and they possess unique photoprotective properties [67,68,69,70,71]. Some commonly used natural products in sunscreens (Table 2) include flavonoids, polyphenols, terpenoids, melanins, and MAAs, which have been found to have photoprotective and other biological properties [19,67,68,72]. MAAs are highly profuse secondary metabolites found in many marine, freshwater, and terrestrial species [8,9]. Rich sources containing different NPs involved in UV protection are provided, including a number of examples specifically for MAAs (Table 2). More comprehensive details about additional MAAs, their chemical structures, and specific features and resources have been provided in MAA databases and reviews [9,44].

Natural products that are considered UV sunscreens should possess several essential features. One of these key elements is the ability to absorb UV radiation effectively and provide broad-spectrum protection; this means that the compounds should be able to absorb both UVA and UVB radiation. Additionally, the stability of the natural products in the presence of UV light is crucial, as any degradation or decomposition of the compound can lead to a loss of protection [73,74]. The ability to demonstrate a high efficacy even at low concentrations is also desirable as it allows for the practical incorporation of the compounds into sunscreen formulations. Safety is another critical factor that should be considered, as the candidates should not cause adverse effects on the skin, such as cytotoxicity or irritations, and should demonstrate minimal permeation into the systemic circulation [75]. When exploring the photoprotective properties of NPs, various types of models were used, including in vitro human skin keratinocytes (HaCaT cells) when assessing quercetin [76], in vivo mouse models when testing myricetin [77], and cell-free assays when evaluating tannic acid bioactivities [78]. Mycosporine-glycine antioxidant activity was assessed using the DPPH radical scavenging assay to investigate in vivo ROS quenching processes [79], while in vitro human keratinocytes were used for the evaluation of the antioxidant activity of palythine [80].

The solubility of the NP candidate in the solvent system used for the sunscreen formulation is essential to ensure that it can be easily incorporated and evenly distributed throughout the product [81]. MAAs have a high water solubility that allows for their distribution within the cell cytoplasm. There were concerns regarding whether MAA water solubility could present an additional challenge when using these molecules within sunscreen formulations for UVR protection during aquatic activities [18]. However, the main component of all sunscreens is water, and it is critical to have appropriate solubilization of these UV filters [82]. For example, other sunscreen formulations successfully used water-soluble synthetic UV filters such as benzophenone-4 [83], indicating that the hydrophilic nature of MAAs should not prevent their use in sunscreen products.

In summary, natural products offer a promising avenue for the development of safe and effective UV sunscreens. By possessing key features such as UV-absorbing properties, broad-spectrum protection, photostability, high efficacy, safety, and solubility, natural product compounds can be considered viable candidates for sunscreen formulations. Nonetheless, a single compound may not be sufficient for adequate skin protection. Instead, it is recommended to consider a combination of various natural substances [70]. Although numerous products with natural ingredients are readily available in the market, none so far have fulfilled all consumer expectations. Hence, the primary focus of new product development should be on addressing these gaps by aiming to identify and characterize more natural product candidates that can help provide effective sun protection and minimize potential health risks.

**Table 2:** Photoprotective natural products with the potential to be used as sunscreen agents due to their UV-absorbing capacities and/or antioxidant properties capable of reducing UV-mediated damage.

UV-Protective Natural Products		Chemical Structure	Key Features/Bioactive Properties	Source of the Compounds
Flavonoids	Quercetin (C_15_H_10_O_7_)	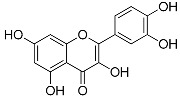	UV-absorbing, antioxidant-scavenging reactive oxygen species induced by UVA and UVB radiation [76,84]	Fruits and vegetables [85,86,87]
	Apigenin (C_15_H_10_O_5_)	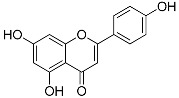	UV-absorbing, antioxidant against UVA and UVB radiation [88]	Parsley, celery, celeriac, basil, chamomile tea [89,90,91]
	Myricetin (C_15_H_10_O_8_)	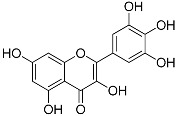	UV-absorbing, suppressing UVB-induced wrinkle formation [77]	Fruits, vegetables, tea, red wine [92]
	Kaempferol (C_15_H_10_O_6_)	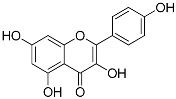	UV-absorbing, antioxidant [93]	Fruits and vegetables: grapes, tomatoes, broccoli, spinach [94]
	Taxifolin (C_15_H_12_O_7_)	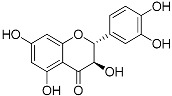	UVA- and UVB-protective [84,95], antioxidant [96]	Citrus fruits and onion [96]
Polyphenols	(−)-Epigallocatechin gallate (C_22_H_18_O_11_)	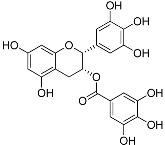	UV-absorbing [97], antioxidant [98]	Green tea [99]
	Tannic acid (C_76_H_52_O_46_)	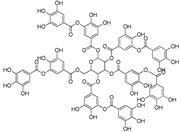	UV-absorbing, antioxidant [78]	All aerial plant tissues [100]
	Resveratrol (C_14_H_12_O_3_^)^	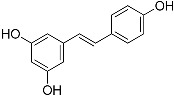	UV-absorbing [101], antioxidant [102]	Grapes, apples, wine, peanuts, and soy [103,104]
	Curcumin (C_21_H_20_O_6_)	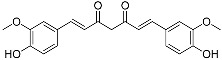	UV-absorbing [105] anti-inflammatory [106]	Plant *Curcuma longa* [90]
Terpenoids	α-Tocopherol (C_29_H_50_O_2_)	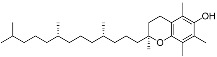	UV-absorbing (385 nm) [107], antioxidants [108]	Vegetable oils, nuts, and whole grains [109]
	Astaxanthin (C_40_H_52_O_4_)	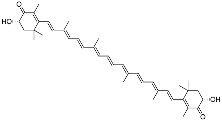	Antioxidants, prevented UVA-mediated DNA damage [110]	Fungi, bacteria, algae, crustaceans, and some fishes [111,112]
Mycosporines-like amino acids	Mycosporine-glycine (C_10_H_15_NO_6_)	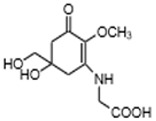	UV-absorbing, antioxidants [79,113]	Cyanobacteria *Chlorogloeopsis* sp. PCC6912 [114]; *Gloeocapsa* sp. [115]; *Nostoc commune* [116] Macroalgae *Acanthophora specifera* [22,117], *species from genus Bostrychia* [118], and *Devaleraea* [119] Arthropoda, Molluscs, Cnidaria, Echinodermata, Protochordata, Phytoplankton, Nemertinea, Porifera, etc. [9]
	Shinorine (C_13_H_20_N_2_O_8_)	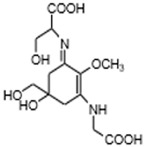	UV-absorbing, antioxidants [38,120]	Cyanobacteria (*Chlorogloeopsis* sp. PCC6912 [114]; *Gloeocapsa* sp. [115]; *Nostoc commune* [53]) Macroalgae *Acanthophora specifera* [22], species from the genus *Asparagopsis* [56,57,58] and *Bostrychia* [118], and *Devaleraea ramentacea* [119] Arthropoda, Molluscs, Cnidaria, Echinodermata, Protochordata, Phytoplankton, Nemertinea, Porifera etc. [9]
	Porphyra-334 (C_14_H_22_N_2_O_8_)	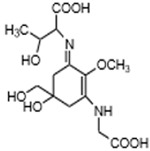	UV-absorbing, antioxidants [38,120]	Cyanobacteria *Nostoc harveyana* [52] Macroalgae species from the genus *Bostrychia* [118] and *Porphyra* [117] *Devaleraea ramentacea* [119] Arthropoda, Molluscs, Cnidaria, Echinodermata, Protochordata, Phytoplankton Nemertinea, Porifera etc. [9]
	Mycosporine-2-glycine (C_12_H_18_N_2_O_7_)	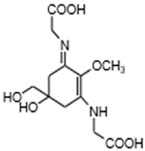	UV-absorbing, antioxidants [42]	Cyanobacteria *Euhalothece* sp. LK-1 [121] and *Aphanothece halophytica* [122] Sea anemone *Anthopleura elegantissima* [123], dinoflagellate *Maristentor dinoferus* [124] Molluscs, Cnidaria and others [9]
	Palythine (C_13_H_20_N_2_O_5;_)	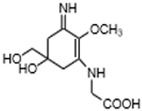	UV-absorbing, antioxidants [80]	Macroalgae *Acanthophora specifera* [22,117], *Bostrychia species* [118] Phytoplankton, Porifera Chordata [9]

## 4. Limitations and Challenges in Using UV-Absorbing MAAs in Sunscreens

Commercially used sunscreens contain synthetic organic and inorganic UVR filters covering a broad range of UVR spectra [18]. Organic UV filters are capable of absorbing UVR, accompanied by inorganic filters such as titanium dioxide (TiO2) and zinc oxide (ZnO), which are also responsible for UVR reflection and scattering [18]. As many synthetic UV filters have a low photostability and a negative effect on the environment, the search for an improved, new generation of UV filters has been ongoing over the last decade [63]. Hundreds of compounds with photoprotective properties were explored, and natural products gained a special interest due to the shift towards environmental safety and raising consumer consciousness [63,125]. MAAs are photoprotective NPs with a supreme potential for use in the new generation of sunscreens due to their abundant presence in marine species, broad UV absorption spectra, and additional roles in actions against osmotic, thermal, and desiccation stress [19,20,30]. MAAs stand out from other photoprotective NPs because of their additional therapeutic properties and ability to accomplish antioxidant, anti-cancer, and anti-inflammatory activities [45]. In addition, the unique ability to perform activation of the Keap1-Nrf2-ARE pathway stimulates cytoprotective gene expression, which is essential for reducing UV-induced damage [38]. Furthermore, MAA mycosporine-2-glycine downregulated gene expression of oxidative stress-induced Cu/Zn-superoxide dismutase 1 and catalase acting on the molecular level and attenuating UVR cellular damage [42].

**Figure 1:**
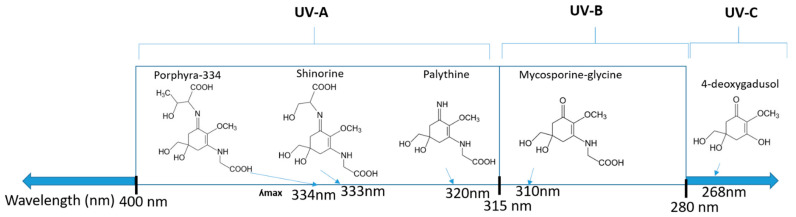
Chemical structures of mycosporine-like amino acids and their maximum absorbance values placed on the UV wavelength scale.

Some of the most abundant MAAs found in nature, including shinorine, porphyra-334, palythine, and mycosporine-glycine (Table 2), have been used in several cosmetic applications as natural sun protection agents. There are 48 patents reported covering the production and/or specific use of MAAs [44]. The sunscreen product Helioguard ^®^365 contains two MAAs, shonorine and porphyra-334, which were isolated from the red seaweed *Porphyra umbilicalis* [126]. In Helionori^®^, palythine, porphyra-334, and shinorine, also isolated from *P. umbilicalis,* were used in the sunscreen formulation. However, the proportion of MAAs in these formulations was low and also provided more protection in the UVA region (Figure 1) [44]. This critical gap would need to be covered by other UV filters as the skin damage coming from UVB is 1000x higher than that from UVA [127]. As the most abundant MAAs have absorption maxima in UVA, including another abundant MAA with a UVB absorption maximum (such as mycosporine-glycine) into the sunscreen formulations would be highly beneficial. Furthermore, to obtain an improved photoprotective capacity, an increase in the extracted MAA concentration dry weight (DW) content is needed [18,128]. An exponential rise in sun protection factor (SPF) values was observed with the increase in the MAA content (containing palythine, asterina-330, shinorine, porphyra-334, and palythinol) isolated from the red algae *Hydropuntia cornea* and *Gracilariopsis longissima,* reaching SPF 7.5 at the highest MAA yield (13.9 mg DW of algae per cm^−2^) [128]. The total MAA content in *H. cornea* was 0.8 ± 0.1 mg MAAs g^−1^ DW, with the main MAA being palythinol (49.2% of the total MAAs), while in *G. longissima*, there was 1.6 ± 0.1 mg MAAs g^−1^ DW with dominant MAA Asterina-330 (42.9% of all MAAs). Furthermore, in red macroalgae *Gracilaria gracilis*, which was exposed to different light conditions, the highest total MAA content was reached under UV lights (133.03 ± 41.54 mg MAAs g^−1^ DW), demonstrating an increase of 162% compared to the control (cultures exposed to actinic yellow light at 590 nm) [129]. Interestingly, light quality influenced the composition of MAAs, with the highest content of palythine accumulated in the presence of red light (620–670 nm), while the addition of UV (280–400 nm) or blue (400–450 nm) resulted in the highest content of shinorine. Others have also observed the impact of the modulation of UV and visible light on MAA yield and profile [130,131,132,133]. Obviously, obtaining a higher MAA content and specific MAA profiles will improve SPF levels, allowing them to be more competitive with other synthetic UV filters. Manipulating the light conditions further can reveal improved ways to direct the MAA production towards desired MAA compounds in more controlled ways in the future.

To enable the widespread use of MAAs, it is also important to successfully apply heterologous expression systems [9]. The limited success here was one of the reasons preventing extensive industry use of MAAs, combined with low extraction yields from natural resources. These existing limitations prompted attempts at chemical synthesis, producing a number of synthetic MAA analogues, which are promising candidates for use in commercial products but have resulted in restricted biological activities compared to the variety of MAAs [10].

## 5. Investigation of the Efficacy of Natural Products for Use as Sunscreens

Natural products, or novel compounds inspired by natural products, hold enormous potential for developing new sunscreens due to their wide structural diversity. The testing methods for the assessment of the effectiveness of the photoprotection of sunscreens applied topically are indicated by a regulatory norm (ISO24444:2019), which determines the sun protection factor (SPF) [29]. However, the true effectiveness of sunscreens is more variable in practice due to differences in skin phenotype (Table 1), geographical location, meteorological considerations, and, most importantly, the amount and frequency of sunscreen application. A key value for determining SPF is the minimal erythemal dose (MED), defined as the dose of solar radiation that produces sunburn [29]. In fact, the ratio of MED with and without sunscreen is the number reflected by the SPF value. An additional important factor is the UVA protection factor (UVA-PF), obtained on UVA transmittance from in vitro measurements (ISO24443:2020). While formal assessment of the efficacy of new sunscreens is well defined, the development of agents with potential photoprotective properties can occur through a series of pre-clinical models [30].

Several models are available to assess the potential for and efficacy of natural products, such as sunscreens, for human use (Table 3). Each of these different models has advantages and disadvantages. The simplest and most cost-effective model is in vitro testing of the compound for protection against UV-induced cell killing or other biological output in cultured human keratinocytes or immortalized keratinocyte lines (such as HaCaT) [134,135]. While these in vitro assays are easy to perform with equipment usually found in a majority of laboratory settings, they do not reflect the true situation in which keratinocytes are protected by the overlying stratum corneum, which is the outermost layer of the epidermis [136]. Previous work has aimed to address these differences and use a more realistic solar spectrum than that seen at the basal layer of the skin [137]. Other in vitro models use reconstructed human skin or human skin explants. The reconstructed human skin model relies on cultured primary keratinocytes and fibroblasts self-organizing into a structure reminiscent of normal skin [138,139]. Melanocytes, the pigment-producing cells within the skin, may also be included. While this reconstructed skin is closer to human skin, the strata are generally thinner, and the model can be technically challenging. Human skin explants are realistic models [140], but they must be used soon after being excised and require human ethics approval. All in vitro models have the additional advantage of a reduction in the number of animals used for research purposes.

A number of in vivo models have been used to test the photoprotection capacity of sunscreens. Prevention of the ear swelling response of the hairless albino mouse using sunscreen was the work-horse model for a period of time [141]. Other murine models, including the Skh:Hr1 mouse [142], the HGF/SF mouse [143], and various transgenic animals (i.e., BRAF V600E [144], XPA knockout [145]) have also been used. Depending on the model, the readout showing the effect of the compound being tested for photoprotection can be easy and rapid, as in the case of the ear swelling model, or experimentally laborious, as in the case of detecting Tp53 induction or DNA damage in the BRAF or HGF/SF mice, respectively. However, in general, mouse skin lacks the layers of strata that are seen in human skin. Therefore, mouse skin does not represent a perfect model for determining sunscreen efficacy. An additional model that has been used is the porcine skin model. Various different sites on the pig have been tested, including the ear and back [146,147]. Again, the layers of strata vary significantly across the pig, so care must be taken to match the human skin as closely as possible. The porcine models are generally hard to access and expensive in comparison to the murine models. Testing novel sunscreens is most appropriate using the skin of healthy human volunteers [148]. However, accessing volunteers for the testing of new sunscreens requires human ethics approval, clinical support, and specialist equipment that is validated and safe for use in humans, leading to expensive testing. Clearly, the use of pre-clinical models has a place in the development of novel, natural product sunscreens.

**Table 3:** Advantages and disadvantages of various models to test novel sunscreen efficacy.

	Model	Advantages	Disadvantages
In vitro	Cell culture—primary/immortalized keratinocytes [134,135]	Inexpensive Equipment found in most laboratories	Unrealistic—no strata Difficult to translate data to alternative models
	Reconstructed skin [138,139]	Mixture of cells	Unrealistic—thin strata Technically challenging
	Skin explants [140]	Real human skin	Requires ethics approval Immediate use
In vivo	Murine models [141,142,143]	Relatively straightforward Well characterized	Unrealistic—thin strata Output can be time-consuming
	Porcine models [146,147]	More realistic	Variable strata depending on the site Expensive, difficult to access
	Human volunteers [148]	Realistic	Requires ethics approval Clinical support Specialist equipmen Expensive

## 6. Genetics of Marine Organisms Producing MAAs

MAAs are heterologous groups of over 30 small (<400 Da), colorless, hydrophilic molecules with a core structure made of a cyclohexanone or cyclohexenimine ring that is conjugated with an additional radical group [12,149,150,151]. These additional groups added to the MAA core, including further carboxylation and demethylation changes, may alter MAA UV absorption properties [12]. The diversity in the MAAs’ composition and yield, including the UV-absorbing capacity, was detected in various species [12,13,22,122]. MAAs are produced via enzymes encoded by genetically diverse complex enzyme pathways. MAA biosynthesis occurs via two pathways, i.e., the shikimate pathway [17] and/or pentose phosphate pathway, leading to the same MAA precursor 4-deoxygadusol (4-DG), known as a direct precursor of MAAs [152,153]. From 4-DG, MAA biosynthesis leads to the creation of different primary and secondary MAAs (Figure 1) [153,154].

Using genome mining approaches [45,155,156,157], the discovery of MAA biosynthetic pathways occurred through the identification of the gene counterparts in different Gram-positive bacteria [154], cyanobacteria [150,152,156,158,159], and microalgae *Symbiodiniaceae* [160,161]. All species capable of MAA synthesis were found to have highly similar sequences corresponding to genes from the MAA shikimate or pentose phosphate pathways [160]. The presence of genetic diversity within genes from MAA pathways among marine species indicated the potential for the differential regulation of MAA biosynthetic processes [19,150,156]. Species capable of generating MAAs contained genes from the *mys* clyster, including dehydrogenase (encoded gene *dehydroquinate synthase*; DHQS) or a homolog of 2-epi-5-epi-valiolone synthase (EVS; gene *mys*A) and the oxidoreductase-encoded gene *O-methyltransferase* (O-MT; gene *mys*B), needed for the production of 4-DG [152]. Similarly, in the cyanobacterium, *Anabaena variabilis* ATCC 29413, the existence of certain *mys* genes resulted in the capacity to generate specific MAAs [152]. For example, the presence of nonribosomalpeptide synthetase (NRPS; encoded by gene *mys*E) enabled the production of mycosporine-glycine, while the presence of a full 4-gene cluster that included the ATP-grasp homolog gene (*mys*C) led to the production of shinorine. However, there are levels of variability detected in the order of the genes encoding the enzymes from the MAA biosynthetic cluster [150,154]. In addition, some species were with or without *mys*E and D-Ala-D-Ala ligase (encoded by gene *mys*D) from the Nostoc-type *mys* cluster [162] and were also characterized by multiple copies of specific genes within MAA biosynthetic gene clusters (BGCs) [45,154,156]. The link between the genetic variability of MAA BGC and the functional profile of synthesized MAAs was recently discussed in Brazilian cyanobacteria [150]. However, from 10 analyzed cyanobacterial strains, the only MAAs successfully quantified were shinorine and porphyra 334 and only in two strains, while the levels of these MAAs were influenced by the media used and UV conditions [150]. Simultaneous exposure to photosynthetically active radiation (PAR: 400–700 nm) and UV lights (16 h PAR + UVR: 8 h dark photocycle over 12 days) resulted in the successful induction of MAA production in Antarctic red macroalgae naturally living in shallow waters and the up to 10-fold increase in the MAA yield [163]. Others also reported the variation in the MAA content was impacted by seasonal variation and nutrient conditions [20,25,26,33,122,164,165,166,167,168]. However, a clear link regarding the regulatory processes affecting the MAA biosynthesis, their BGCs up- and down-regulation, and corresponding MAA composition is still missing.

## 7. Conclusions

Commercially available sunscreens containing synthetic UV filters lack photostability and can result in allergic reactions and inadequate skin protection from damaging UVR. Furthermore, these UV filters pollute our environment and negatively affect living organisms’ delicate balance. Therefore, using natural UV filters should be further explored for the future shift towards sustainable green technologies. MAAs are exceptional candidates among these UV-absorbing compounds, offering skin UVR protection and cosmetic benefits while being ecologically sustainable. A better understanding of regulatory processes and conditions impacting MAA biosynthesis via abiotic factors is critical for the improved and controlled production of MAAs in heterologous expression systems or even when harvesting from the natural environment. Utilizing the advanced pharmacological properties of MAAs and their UV protective capacities may provide additional skin sun protection, creating a new generation of environmentally friendly sunscreens. However, multiple challenges remain unresolved. Substantial knowledge gaps still exist, including the best ways to stimulate and regulate MAA biosynthesis to obtain higher yields and produce targeted MAAs absorbing in both the UVA and UVB ranges. Consequently, further studies are needed to enable controlled MAA production in vivo and in vitro and to improve the amalgamation of MAAs in sunscreen formulations to enhance their future use in UVR protection and skin cancer prevention.

## Figures and Tables

**Table 1:** Skin cancer risk based upon Fitzpatrick skin type.

Fitzpatrick Skin Type	Typical Features	Tanning Ability and Sensitivity	Skin Cancer Risk *
I	Unexposed skin: white Eyes: blue or green Frequent freckling Northern European or British	Always burns with minimal UV exposure Peels Never tans	4
II	Unexposed skin: white Eyes: blue, hazel or brown Hair: red, blonds or brown European or Scandinavian	Burns easily Peels Tans minimally	3–4
III	Unexposed skin: fair Eyes: brown; Hair: dark Southern or Central European	Burns moderately Average tanning ability	3
IV	Unexposed skin: light brown Eyes: dark; Hair: dark Mediterranean, Asian, or Latino	Burns minimally Tans easily	2
V	Unexposed skin: brown Eyes: dark; Hair: dark East Indian, Native American, Latino, or African	Rarely burns Tans easily and substantially	1
VI	Unexposed skin: black Eyes: dark; Hair: dark African or Aboriginal	Almost never burns Tans readily and profusely	0.5

Note: * 1 is the lowest risk, 4 is the highest risk.

## Data Availability

Not applicable.
